# Effect modification by fatty liver in the association between triglyceride-Glucose index and new-onset hypertension: a large health-examination cohort study

**DOI:** 10.3389/fendo.2026.1897050

**Published:** 2026-08-31

**Authors:** Yuzhan Lin, Lifeng Wu, Shaorong Yan, Ruixue Sun

**Affiliations:** Department of Clinical Laboratory, The Third Affiliated Hospital of Wenzhou Medical University, Ruian, Zhejiang, China

**Keywords:** cohort study, fatty liver, insulin resistance, interaction, new-onset hypertension, triglyceride-glucose index

## Abstract

**Background:**

The triglyceride-glucose (TyG) index is a simple surrogate marker of insulin resistance. Whether baseline fatty liver modifies the association between TyG and incident hypertension has not been evaluated in large longitudinal cohorts.

**Methods:**

This retrospective cohort study included 66,664 adults without hypertension at baseline who underwent routine health examinations and follow-up. The TyG index was calculated from fasting triglyceride and fasting glucose concentrations and analyzed as both a continuous and categorical variable. Baseline fatty liver status was assessed by abdominal ultrasonography. Participants were followed for a median of 2.37 years (interquartile range, 1.69-3.21 years). Multivariable Cox proportional hazards models adjusted for age, sex, marital status, ethnicity, body mass index, lifestyle factors, cholesterol measures, and liver enzymes were used to estimate hazard ratios (HRs) and 95% confidence intervals (CIs). Effect modification by fatty liver status was evaluated using stratified analyses and a TyG-by-fatty liver interaction term. Joint associations of TyG category and fatty liver status with new-onset hypertension were also examined.

**Results:**

During follow-up, 13,949 (20.9%) participants developed new-onset hypertension. Higher TyG was independently associated with new-onset hypertension as a continuous variable (HR 1.37, 95% CI 1.30-1.45) and in the highest versus lowest quartile (HR 1.61, 95% CI 1.49-1.75). Participants with both high TyG and fatty liver had the highest risk (HR 1.63, 95% CI 1.52-1.76), and fatty liver significantly modified the TyG-hypertension association (P for interaction <0.001). These associations remained materially unchanged in sensitivity analyses with additional adjustment for family history of hypertension, diabetes status, kidney function, and baseline blood pressure.

**Conclusions:**

Higher baseline TyG and fatty liver were associated with an increased risk of new-onset hypertension. Baseline fatty liver modified the association between TyG and new-onset hypertension. These findings may help inform the interpretation of TyG-related future hypertension risk according to baseline fatty liver status among adults undergoing routine health examinations.

## Introduction

Hypertension remains one of the leading modifiable contributors to cardiovascular morbidity and mortality worldwide (1, 2). According to the 2025 WHO global report, an estimated 1.4 billion adults aged 30–79 years worldwide were living with hypertension in 2024, and only 23% had their blood pressure controlled (3). Identifying simple metabolic markers that can stratify future hypertension risk may help detect high-risk individuals before blood pressure becomes clinically elevated.

Insulin resistance is closely linked to dyslipidemia, obesity, hepatic steatosis, vascular dysfunction, sympathetic activation, and sodium retention, all of which can contribute to blood pressure elevation (4, 5). The triglyceride-glucose (TyG) index, calculated from fasting triglyceride and glucose levels, was proposed as an accessible surrogate for insulin resistance and has shown acceptable performance compared with more complex insulin-resistance assessments (6, 7). Because TyG integrates two routinely measured components of lipid and glucose metabolism, it may also reflect broader glucose-lipid metabolic disturbance in health-examination populations. Growing epidemiological evidence has linked higher TyG with incident hypertension and other cardiometabolic outcomes (8, 9).

Ultrasonography-detected fatty liver is another common metabolic condition that reflects hepatic lipid accumulation and systemic metabolic dysregulation (10, 11). Prior studies and meta-analyses have suggested that fatty liver is associated with an increased risk of hypertension, while TyG is also closely related to hepatic steatosis and insulin resistance-related metabolic abnormalities (12, 13). Although TyG and fatty liver are closely related, they may capture different manifestations of metabolic dysfunction. Whether baseline fatty liver modifies the association between TyG and new-onset hypertension remains unclear. Given the potential differences in metabolic background between individuals with and without fatty liver, understanding the role of fatty liver as a modifier may be important for interpreting TyG-related risk of new-onset hypertension.

Therefore, this study aimed to investigate the association between baseline TyG and new-onset hypertension in a large health-examination cohort and to evaluate the potential effect modification by baseline fatty liver status. Because an elevated TyG index may more clearly capture early glucose-lipid metabolic disturbance before hepatic steatosis is detectable by ultrasonography, we hypothesized that higher TyG would be associated with an increased risk of new-onset hypertension and that this association would be stronger among participants without fatty liver.

## Methods

### Ethics statement

This study was approved by the Institutional Review Board of the Third Affiliated Hospital of Wenzhou Medical University (YJ2026011). The requirement for informed consent was waived by the review board because of the retrospective nature of the study and the use of fully anonymized data, which posed no identifiable risk to participants. All study procedures were conducted in accordance with the ethical principles of the Declaration of Helsinki.

### Study design and data source

This retrospective cohort study was reported in accordance with the Strengthening the Reporting of Observational Studies in Epidemiology (STROBE) Statement and used data from adults who underwent annual physical examinations at the Third Affiliated Hospital of Wenzhou Medical University between January 1, 2021, and December 31, 2025. The dataset included demographic characteristics, physical examination findings, laboratory measurements, abdominal ultrasonography findings, and questionnaire-based lifestyle information collected as part of routine clinical practice. Individual examination records were linked using a unique identification code. Baseline was defined as each participant’s first health examination during the study period, and follow-up examinations were defined as any subsequent health examinations after baseline.

### Study population

**Figure 1:**
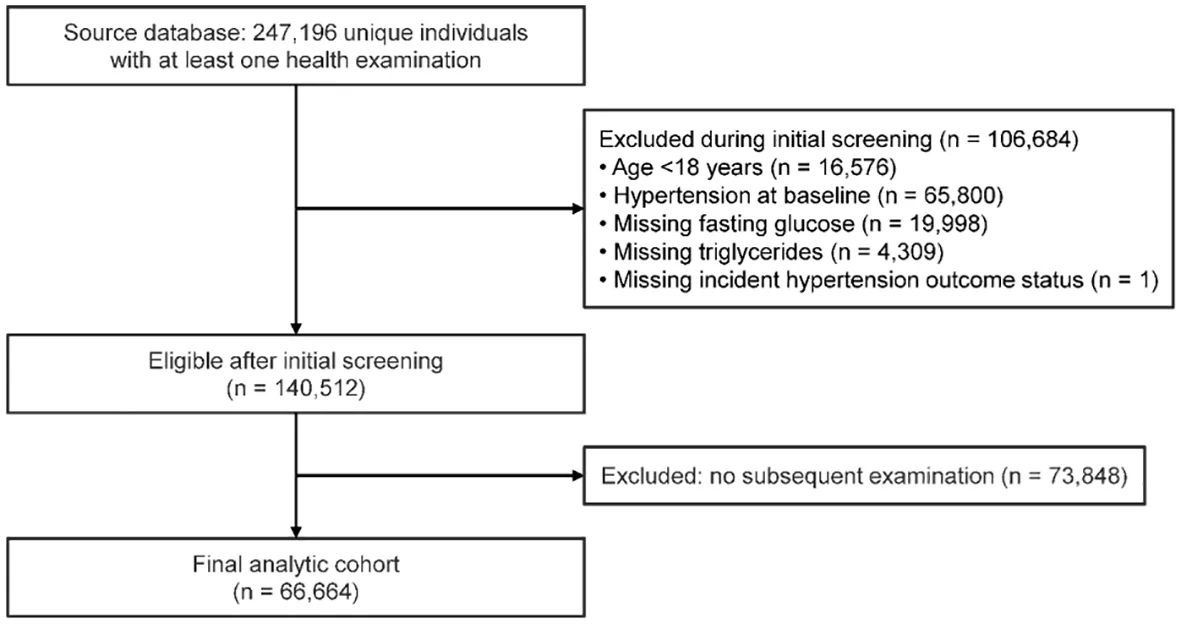
Flowchart of participant selection. Among 247,196 unique individuals with at least one health examination, 79,905 underwent at least one subsequent examination and 167,291 had only one examination record. After sequential application of the eligibility criteria, 66,664 participants were included in the final analytic cohort.

The source database included 247,196 unique individuals who underwent at least one health examination during the study period. Among them, 167,291 underwent only one examination, whereas 79,905 underwent at least one follow-up examination. Individuals with only one examination could not provide longitudinal follow-up information. After applying the prespecified eligibility and exclusion criteria, 66,664 participants were included in the final analytic cohort (**Figure 1**).

### TyG index assessment

The TyG index was calculated as ln[fasting triglycerides (mg/dL) × fasting plasma glucose (mg/dL)/2] (6). Participants were categorized into quartiles based on the distribution of TyG to describe potential dose-response patterns while retaining adequate numbers of participants and events in each group: Q1 (≤8.16), Q2 (>8.16 to ≤8.557), Q3 (>8.557 to ≤8.989), and Q4 (>8.989).

### Fatty liver assessment

Fatty liver status at baseline was determined from abdominal ultrasonography reports obtained during routine health examination. The examinations were performed and interpreted by ultrasound physicians at the time of the health examination. As the reports were generated before follow-up, the ultrasound physicians were not informed of participants’ subsequent hypertension status or study group. The sonographic diagnosis of fatty liver was based on typical features of hepatic steatosis, including increased hepatic echogenicity or liver brightness, increased hepatorenal echo contrast, deep attenuation of the ultrasound beam, and blurring of intrahepatic vascular structures. Participants were classified as having fatty liver or not having fatty liver according to the ultrasound report (14).

### Outcome assessment

The outcome was new-onset hypertension during follow-up. At each health examination, blood pressure was measured using an automated blood pressure monitor according to the routine examination procedures of the health-examination center. The device was regularly calibrated and maintained by the health-examination center. The systolic and diastolic blood pressure values displayed by the device were recorded for outcome ascertainment. New-onset hypertension was defined as systolic blood pressure ≥140 mmHg or diastolic blood pressure ≥90 mmHg measured at a follow-up health examination, self-reported physician-diagnosed hypertension in the questionnaire, or current use of antihypertensive medications. The blood pressure threshold used was consistent with the 2023 European Society of Hypertension guideline (2). Participants were followed from the baseline health examination until the first follow-up visit at which new-onset hypertension was identified or, for participants without hypertension, until the last available follow-up visit.

### Covariates

Covariates were collected at baseline through health examination records and standardized questionnaires. Demographic variables included age, sex, ethnicity, and marital status. Lifestyle factors included smoking status, alcohol drinking, and physical activity. Clinical and biochemical variables included body mass index, total cholesterol, low-density lipoprotein cholesterol, high-density lipoprotein cholesterol, aspartate aminotransferase, and alanine aminotransferase.

### Statistical analysis

Continuous variables were summarized as mean ± standard deviation, and categorical variables were summarized as number and percentage. Baseline characteristics were compared across TyG quartiles using one-way analysis of variance or Kruskal-Wallis tests for continuous variables and chi-square tests or Fisher’s exact tests for categorical variables. To assess potential selection bias related to follow-up availability, baseline characteristics were also compared between included participants and otherwise eligible individuals without follow-up using P values and standardized mean differences (**Supplementary Table 1**).

Cox proportional hazards models were used to estimate hazard ratios (HRs) and 95% confidence intervals (CIs) for the associations of TyG, fatty liver, and joint TyG-fatty liver exposure groups with new-onset hypertension. Model 1 was adjusted for age and sex. Model 2 was further adjusted for marital status, ethnicity, body mass index, smoking, alcohol drinking, physical activity, total cholesterol, low-density lipoprotein cholesterol, high-density lipoprotein cholesterol, aspartate aminotransferase, and alanine aminotransferase. To assess the robustness of the findings, two extended models were specified. Model 3 added family history of hypertension, baseline diabetes status, and estimated glomerular filtration rate to Model 2. Model 4 further added baseline systolic blood pressure and diastolic blood pressure to Model 3. The proportional hazards assumption was assessed after model fitting using scaled Schoenfeld residuals, with variable-specific and global tests. Multicollinearity among TyG and covariates included in Model 2 was assessed using variance inflation factors.

Missing questionnaire responses for smoking status, alcohol drinking status, and physical activity were coded as an “unknown” category for each variable and retained in the multivariable models. Missing data for the other Model 2 covariates were handled using a complete-case approach, and analyses involving baseline fatty liver status were restricted to participants with available baseline fatty liver data.

Restricted cubic spline models with four knots placed at the 5th, 35th, 65th, and 95th percentiles of the TyG distribution were used to examine the potential nonlinear association between TyG and new-onset hypertension in the overall cohort and according to fatty liver status. Likelihood ratio tests were used to assess the overall association and nonlinearity. To examine whether baseline fatty liver status modified the association between TyG and new-onset hypertension, Cox models were performed stratified by fatty liver status. Multiplicative interaction was assessed by including a product term between continuous TyG and baseline fatty liver status in the Cox model. In addition, HR-based additive interaction measures for high TyG and baseline fatty liver status were calculated, including the relative excess risk due to interaction (RERI), attributable proportion due to interaction (AP), and synergy index (SI), with 95% confidence intervals. To further explore the potential influence of BMI on the observed interaction between TyG and baseline fatty liver, we repeated the fatty liver-stratified Cox analyses within BMI categories (<18.5, 18.5–23.9, 24.0–27.9, and ≥28.0 kg/m²).

To evaluate the joint association of TyG and fatty liver status with new-onset hypertension, TyG was dichotomized using the optimal cut-off value identified by receiver operating characteristic curve analysis based on the Youden index (15). This sample-derived cut-off was used only to define the joint exposure groups and was not intended as a universal clinical threshold. Four joint exposure groups were then defined: low TyG without fatty liver, low TyG with fatty liver, high TyG without fatty liver, and high TyG with fatty liver. The low TyG without fatty liver group was used as the reference group. Kaplan-Meier curves were used to visualize hypertension-free survival across the joint exposure groups, and differences between groups were compared using log-rank tests.

All statistical analyses were performed using R software (version 4.5.2). A two-sided P value <0.05 was considered statistically significant.

## Results

### Study population and baseline characteristics

Among the 247,196 unique individuals assessed for eligibility, 79,905 (32.3%) underwent at least one follow-up examination, whereas 167,291 (67.7%) had only one examination record. After applying the prespecified eligibility and exclusion criteria, 66,664 participants were included in the final analytic cohort (**Figure 1**). During a median follow-up of 2.37 years (interquartile range, 1.69-3.21 years), 13,949 participants (20.9%) developed new-onset hypertension. Baseline characteristics of included participants and otherwise eligible individuals without follow-up are presented in **Supplementary Table 1**, with small SMDs observed for most measured clinical characteristics.

Baseline characteristics according to TyG quartiles are shown in **Table 1**. Participants with higher TyG levels were older and more likely to be male and married. Across increasing TyG quartiles, systolic and diastolic blood pressure, triglycerides, fasting glucose, total cholesterol, body mass index, waist circumference, aspartate aminotransferase, and alanine aminotransferase were higher, whereas high-density lipoprotein cholesterol was lower. The prevalence of fatty liver among participants with available fatty liver data increased markedly across TyG quartiles, from 7.0% in Q1 to 69.1% in Q4. During follow-up, 13,949 participants developed new-onset hypertension, and the proportion increased from 10.1% in Q1 to 33.7% in Q4. Correspondingly, the incidence rate of new-onset hypertension increased across TyG quartiles, from 39.48 per 1,000 person-years in Q1 to 75.85, 111.11, and 146.28 per 1,000 person-years in Q2, Q3, and Q4, respectively (**Table 2**).

**Table 1:** Baseline characteristics of participants according to quartiles of the TyG index.

Variables	Q1 (≤8.16)	Q2 (8.16-8.557)	Q3 (8.557-8.989)	Q4 (>8.989)	P value
Participants, n	20390	18125	15621	12528	
Age, years	38.7 ± 12.3	44.1 ± 13.6	46.9 ± 13.6	48.1 ± 12.6	<0.001
Marital status					<0.001
Unmarried	4268 (21.0%)	2381 (13.2%)	1439 (9.2%)	745 (6.0%)	
Married	16094 (79.0%)	15717 (86.8%)	14163 (90.8%)	11746 (94.0%)	
Sex					<0.001
Male	5042 (24.7%)	7539 (41.6%)	8871 (56.8%)	8862 (70.7%)	
Female	15348 (75.3%)	10586 (58.4%)	6750 (43.2%)	3666 (29.3%)	
TyG index	7.9 ± 0.2	8.4 ± 0.1	8.8 ± 0.1	9.4 ± 0.4	<0.001
Baseline fatty liver status					<0.001
No fatty liver	15863 (93.0%)	11831 (78.8%)	7465 (57.9%)	3135 (30.9%)	
Fatty liver	1190 (7.0%)	3174 (21.2%)	5425 (42.1%)	7024 (69.1%)	
Systolic blood pressure, mmHg	112.6 ± 12.1	116.5 ± 12.2	119.8 ± 11.6	122.3 ± 10.8	<0.001
Diastolic blood pressure, mmHg	67.4 ± 8.5	69.7 ± 8.7	71.8 ± 8.5	73.9 ± 8.2	<0.001
Triglycerides, mmol/L	0.7 ± 0.1	1.1 ± 0.2	1.7 ± 0.3	3.1 ± 2.1	<0.001
Glucose, mmol/L	4.5 ± 0.4	4.7 ± 0.5	4.9 ± 0.7	5.5 ± 1.6	<0.001
Total cholesterol, mmol/L	4.6 ± 0.8	4.9 ± 0.9	5.1 ± 0.9	5.3 ± 1.0	<0.001
LDL-C, mmol/L	2.8 ± 0.8	3.1 ± 0.8	3.4 ± 0.9	3.2 ± 1.0	<0.001
HDL-C, mmol/L	1.5 ± 0.3	1.3 ± 0.3	1.2 ± 0.3	1.0 ± 0.2	<0.001
Aspartate aminotransferase, U/L	21.3 ± 10.6	23.1 ± 12.8	25.2 ± 12.6	28.5 ± 16.2	<0.001
Alanine aminotransferase, U/L	18.6 ± 16.6	23.5 ± 22.0	29.8 ± 25.3	39.4 ± 34.6	<0.001
Serum creatinine, μmol/L	58.8 ± 12.8	63.2 ± 17.7	66.5 ± 15.3	69.1 ± 15.9	<0.001
Uric acid, μmol/L	300.2 ± 74.7	330.3 ± 83.6	362.5 ± 89.7	398.8 ± 96.6	<0.001
Height, cm	161.9 ± 7.5	163.2 ± 8.3	164.6 ± 8.5	166.0 ± 8.3	<0.001
Weight, kg	56.3 ± 9.2	60.5 ± 10.6	64.8 ± 11.1	69.1 ± 11.4	<0.001
Body mass index, kg/m²	21.4 ± 2.6	22.6 ± 2.9	23.8 ± 3.0	25.0 ± 2.9	<0.001
Waist circumference, cm	72.8 ± 7.5	76.6 ± 8.2	80.1 ± 8.3	83.2 ± 8.3	<0.001
Hip circumference, cm	90.5 ± 5.9	91.3 ± 6.3	92.2 ± 6.5	93.0 ± 6.4	<0.001
Alcohol drinking status					<0.001
Never drinker	1826 (92.8%)	1533 (88.9%)	1252 (84.7%)	963 (77.7%)	
Former drinker	5 (0.3%)	5 (0.3%)	8 (0.5%)	9 (0.7%)	
Current drinker	136 (6.9%)	186 (10.8%)	219 (14.8%)	267 (21.5%)	
Smoking status					<0.001
Never smoker	1887 (95.7%)	1610 (92.7%)	1321 (88.8%)	1018 (81.7%)	
Former smoker	4 (0.2%)	8 (0.5%)	9 (0.6%)	16 (1.3%)	
Current smoker	81 (4.1%)	119 (6.9%)	157 (10.6%)	212 (17.0%)	
New-onset hypertension					<0.001
No	18338 (89.9%)	14689 (81.0%)	11388 (72.9%)	8300 (66.3%)	
Yes	2052 (10.1%)	3436 (19.0%)	4233 (27.1%)	4228 (33.7%)	
Physical activity					0.411
No regular exercise	1088 (57.0%)	986 (58.7%)	805 (56.5%)	663 (54.9%)	
Occasional exercise	765 (40.1%)	652 (38.8%)	582 (40.8%)	516 (42.8%)	
Regular exercise	57 (3.0%)	42 (2.5%)	39 (2.7%)	28 (2.3%)	

Values are presented as mean ± standard deviation or n (%). TyG index quartiles were defined using the 25th, 50th, and 75th percentile cut points calculated from the TyG distribution in the final analytic cohort. Because participants with identical TyG values at the cut points were assigned to the same interval, the four groups were not exactly equal in size. The exact group boundaries are shown in the column headings. Percentages for categorical variables were calculated among participants with available data for each variable; missing values were not included in the denominator. Comparisons across TyG index quartiles were performed using one-way analysis of variance for continuous variables and the chi-square test for categorical variables. TyG, triglyceride-glucose; LDL-C, low-density lipoprotein cholesterol; HDL-C, high-density lipoprotein cholesterol.

**Table 2:** Association of the TyG index with new-onset hypertension.

Variable	Participants, n (%) or mean ± SD	Events, n (%)	Person-years	Incidence rate per 1,000 person-years	Unadjusted HR (95% CI), P value	Model 1 HR (95% CI), P value	Model 2 HR (95% CI), P value
TyG index	8.50 ± 0.59	—	—	—	2.05 (2.00–2.10), <0.001	1.58 (1.54–1.62), <0.001	1.37 (1.30–1.45), <0.001
TyG index quartiles							
Q1 (≤8.16)	20,390 (30.6%)	2,052 (10.1%)	51,970.52	39.48	Ref	Ref	Ref
Q2 (8.16–8.557)	18,125 (27.2%)	3,436 (19.0%)	45,302.81	75.85	1.96 (1.86–2.07), <0.001	1.42 (1.35–1.51), <0.001	1.30 (1.22–1.39), <0.001
Q3 (8.557–8.989)	15,621 (23.4%)	4,233 (27.1%)	38,096.81	111.11	2.94 (2.79–3.10), <0.001	1.82 (1.73–1.93), <0.001	1.51 (1.41–1.61), <0.001
Q4 (>8.989)	12,528 (18.8%)	4,228 (33.7%)	28,903.76	146.28	4.02 (3.82–4.24), <0.001	2.27 (2.15–2.40), <0.001	1.61 (1.49–1.75), <0.001

Values are presented as hazard ratios (HRs) and 95% confidence intervals (CIs), with corresponding P values. The outcome was new-onset hypertension. Incidence rates are expressed per 1,000 person-years.

Model 1 was adjusted for age and sex. Model 2 was adjusted for age, sex, marital status, ethnicity, smoking status, physical activity, alcohol drinking status, body mass index, aspartate aminotransferase, alanine aminotransferase, total cholesterol, low-density lipoprotein cholesterol, and high-density lipoprotein cholesterol. The sample size was 66,664 for the unadjusted model and Model 1, and 57,597 for Model 2. Missing questionnaire responses for smoking status, alcohol drinking status, and physical activity were coded as an “unknown” category, whereas missing data for the other Model 2 covariates were handled using a complete-case approach. TyG, triglyceride-glucose index; HR, hazard ratio; CI, confidence interval; Ref, reference.

### Association of TyG with new-onset hypertension

**Figure 2:**
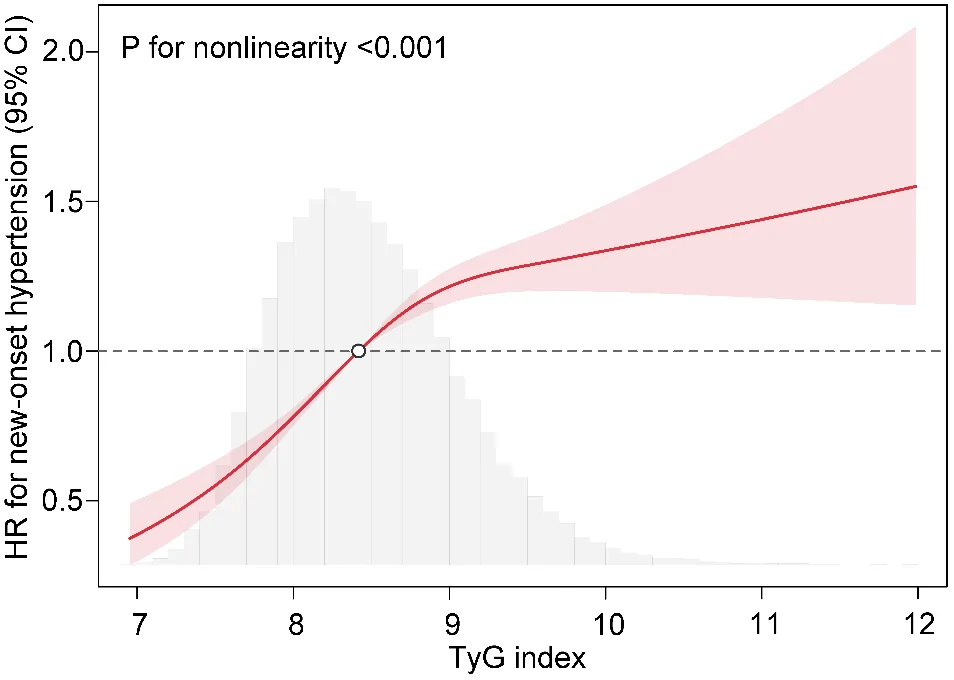
Association between the TyG index and new-onset hypertension. Restricted cubic spline curve showing the association between the triglyceride-glucose (TyG) index and the risk of new-onset hypertension. The model used four knots placed at the 5th, 35th, 65th, and 95th percentiles of the TyG distribution. Hazard ratios were referenced to a TyG value of 8.42. The P value for nonlinearity was <0.001. The solid line represents the hazard ratio (HR), and the shaded area represents the 95% confidence interval (CI). The histogram indicates the distribution of the TyG index. The model was adjusted for age, sex, marital status, ethnicity, body mass index, smoking status, alcohol drinking status, physical activity, total cholesterol, low-density lipoprotein cholesterol, high-density lipoprotein cholesterol, aspartate aminotransferase, and alanine aminotransferase.

Higher TyG was associated with an increased risk of new-onset hypertension (**Table 2**). When TyG was analyzed as a continuous variable, each one-unit increase in TyG was associated with a higher risk of new-onset hypertension in the fully adjusted model (HR, 1.37; 95% CI, 1.30-1.45; P<0.001). Similar associations were observed when TyG was analyzed by quartiles. Compared with participants in Q1, the fully adjusted HRs for new-onset hypertension were 1.30 (95% CI, 1.22-1.39) for Q2, 1.51 (95% CI, 1.41-1.61) for Q3, and 1.61 (95% CI, 1.49-1.75) for Q4. Restricted cubic spline analysis showed a nonlinear association between TyG and new-onset hypertension (P for nonlinearity <0.001; **Figure 2**). The proportional hazards assumption for TyG was not rejected by the scaled Schoenfeld residual test (P = 0.151). No evidence of severe multicollinearity was observed in Model 2.

### Association of fatty liver with new-onset hypertension

Among participants with available fatty liver status, 16,813 (30.5%) had fatty liver at baseline (**Table 3**). Baseline fatty liver was associated with a higher risk of new-onset hypertension. After multivariable adjustment, participants with fatty liver had a 26% higher risk of new-onset hypertension than those without fatty liver (HR, 1.26; 95% CI, 1.19-1.33; P<0.001).

**Table 3:** Association between baseline fatty liver status and new-onset hypertension.

Variable	Participants, n (%)	Unadjusted HR (95% CI), P value	Model 1 HR (95% CI), P value	Model 2 HR (95% CI), P value
Baseline fatty liver status				
No fatty liver	38,294 (69.5%)	Ref	Ref	Ref
Fatty liver	16,813 (30.5%)	2.45 (2.37-2.54), <0.001	1.75 (1.69-1.82), <0.001	1.26 (1.19-1.33), <0.001

Values are presented as hazard ratios (HRs) and 95% confidence intervals (CIs), with corresponding P values. The outcome was new-onset hypertension. Analyses were restricted to participants with available baseline fatty liver status.

Model 1 was adjusted for age and sex. Model 2 was adjusted for age, sex, marital status, ethnicity, body mass index, smoking status, alcohol drinking status, physical activity, total cholesterol, low-density lipoprotein cholesterol, high-density lipoprotein cholesterol, aspartate aminotransferase, and alanine aminotransferase. The sample size was 55,107 for the unadjusted model and Model 1, and 41,984 for Model 2. Missing questionnaire responses for smoking status, alcohol drinking status, and physical activity were coded as an “unknown” category, whereas missing data for the other Model 2 covariates were handled using a complete-case approach. Ref, reference.

### Joint association of TyG category and fatty liver status

**Figure 3:**
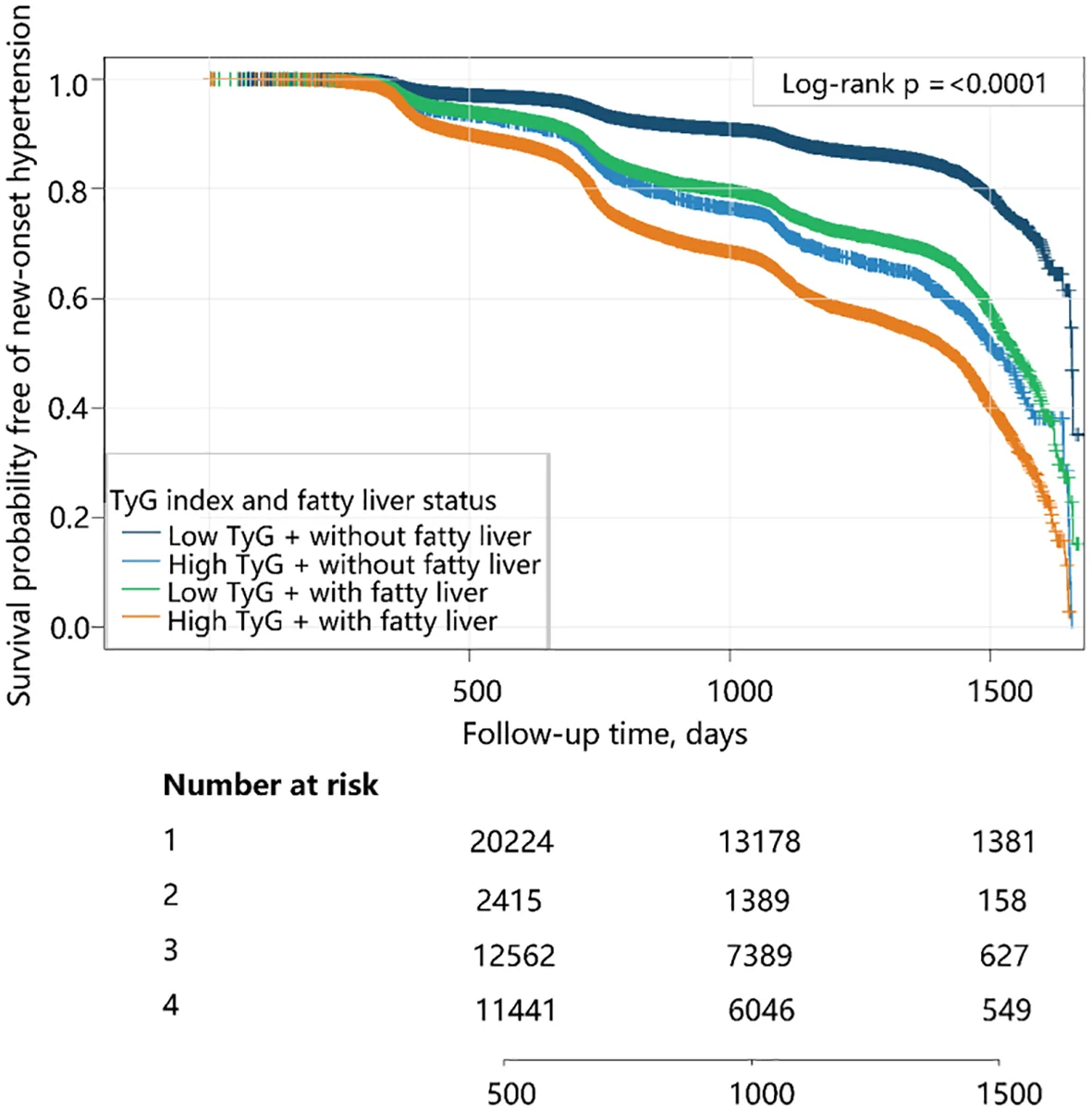
Kaplan-Meier curves for new-onset hypertension according to TyG index and fatty liver status. Kaplan-Meier curves showing the probability of remaining free from new-onset hypertension according to TyG index category and baseline fatty liver status. Differences between groups were compared using the log-rank test. The number-at-risk table is shown below the curves.

For the joint exposure analysis, TyG was dichotomized at the receiver operating characteristic curve-derived cutoff of 8.40. This cutoff had an area under the curve of 0.659 (95% CI, 0.655-0.664), a sensitivity of 72.1%, a specificity of 52.3%, and a Youden index of 0.244 for new-onset hypertension. The joint association of TyG category and fatty liver status with new-onset hypertension is shown in **Table 4**. Compared with participants with low TyG and no fatty liver, the fully adjusted HRs for new-onset hypertension were 1.45 (95% CI, 1.30-1.60) for low TyG with fatty liver, 1.42 (95% CI, 1.33-1.52) for high TyG without fatty liver, and 1.63 (95% CI, 1.52-1.76) for high TyG with fatty liver. HR-based additive interaction analyses did not provide evidence of positive additive interaction between high TyG and baseline fatty liver status (**Supplementary Table 5**). Kaplan-Meier curves showed significant differences in hypertension-free survival across the four joint exposure groups, with the lowest hypertension-free survival observed among participants with both high TyG and fatty liver (log-rank P<0.001; **Figure 3**).

**Table 4:** Joint association of TyG index and fatty liver status with new-onset hypertension.

Variable	Participants, n (%)	Unadjusted HR (95% CI), P value	Model 1 HR (95% CI), P value	Model 2 HR (95% CI), P value
Joint TyG index and fatty liver status				
Low TyG + no fatty liver	23,479 (42.6%)	Ref	Ref	Ref
Low TyG + fatty liver	2,796 (5.1%)	2.74 (2.54-2.97), <0.001	1.93 (1.78-2.08), <0.001	1.45 (1.30-1.60), <0.001
High TyG + no fatty liver	14,815 (26.9%)	2.31 (2.20-2.43), <0.001	1.60 (1.52-1.68), <0.001	1.42 (1.33-1.52), <0.001
High TyG + fatty liver	14,017 (25.4%)	3.84 (3.67-4.03), <0.001	2.35 (2.23-2.46), <0.001	1.63 (1.52-1.76), <0.001

Values are presented as hazard ratios (HRs) and 95% confidence intervals (CIs), with corresponding P values. The outcome was new-onset hypertension. The reference group was participants with low TyG index and no fatty liver. High TyG was defined as a TyG index >8.40, based on the receiver operating characteristic curve-derived cutoff.

Model 1 was adjusted for age and sex. Model 2 was adjusted for age, sex, marital status, ethnicity, body mass index, smoking status, alcohol drinking status, physical activity, total cholesterol, low-density lipoprotein cholesterol, high-density lipoprotein cholesterol, aspartate aminotransferase, and alanine aminotransferase. The sample size was 55,107 for the unadjusted model and Model 1, and 41,984 for Model 2. Missing questionnaire responses for smoking status, alcohol drinking status, and physical activity were coded as an “unknown” category, whereas missing data for the other Model 2 covariates were handled using a complete-case approach. TyG, triglyceride-glucose index; HR, hazard ratio; CI, confidence interval; Ref, reference.

### Effect modification by fatty liver status

**Figure 4:**
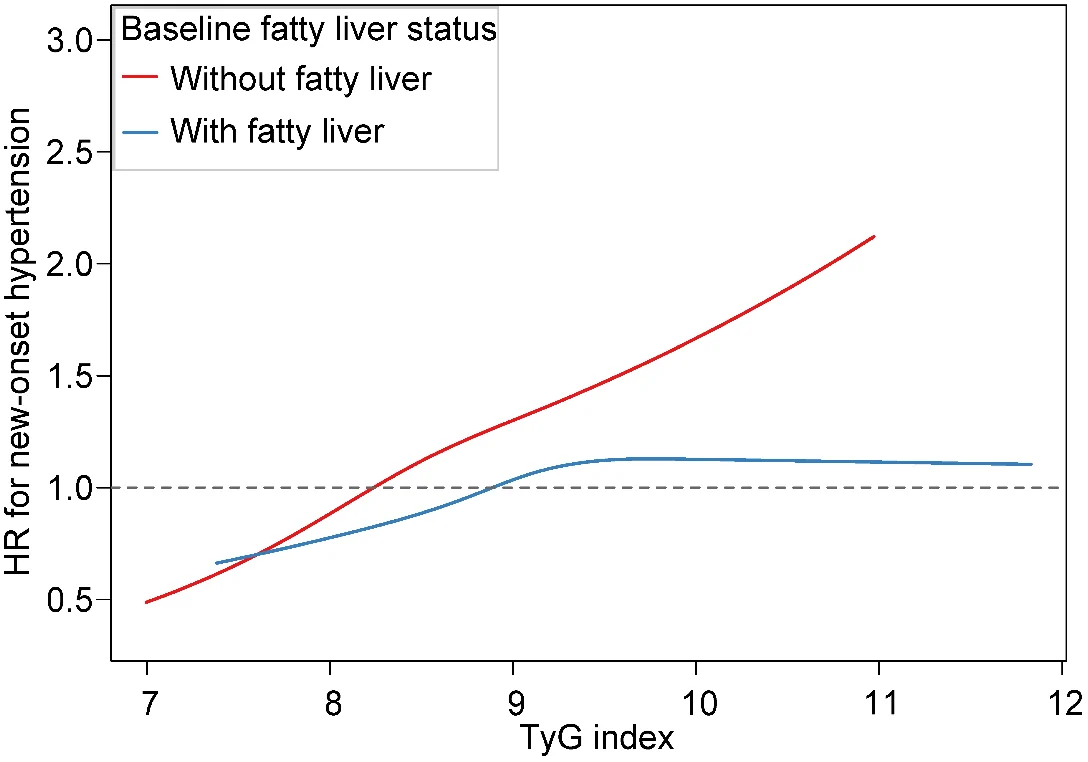
Association between the TyG index and new-onset hypertension stratified by baseline fatty liver status. Restricted cubic spline curves showing the association between the TyG index and the risk of new-onset hypertension stratified by baseline fatty liver status. The solid lines represent hazard ratios for participants with and without fatty liver. The horizontal dashed line indicates an HR of 1.0. The models were adjusted for age, sex, marital status, ethnicity, body mass index, smoking status, alcohol drinking status, physical activity, total cholesterol, low-density lipoprotein cholesterol, high-density lipoprotein cholesterol, aspartate aminotransferase, and alanine aminotransferase.

The association between TyG and new-onset hypertension differed by baseline fatty liver status (**Table 5**). In the fully adjusted model, TyG was more strongly associated with new-onset hypertension among participants without fatty liver (HR, 1.46; 95% CI, 1.33-1.59; P<0.001) than among those with fatty liver (HR, 1.22; 95% CI, 1.12-1.33; P<0.001), with a significant multiplicative interaction between continuous TyG and baseline fatty liver status (P for interaction <0.001). Stratified restricted cubic spline curves showed a steeper increase in hypertension risk with increasing TyG among participants without fatty liver, whereas the curve was comparatively flatter among participants with fatty liver (**Figure 4**). In BMI-stratified sensitivity analyses, the TyG-hypertension association remained stronger among participants without fatty liver in the normal-weight and overweight categories, supporting the persistence of this effect modification across BMI categories (**Supplementary Table 2**). The associations of TyG and fatty liver, their joint exposure, and the TyG-by-fatty liver interaction remained consistent after additional adjustment for family history of hypertension, baseline diabetes status, and estimated glomerular filtration rate, and after further adjustment for baseline systolic and diastolic blood pressure (**Supplementary Table 3** and **S4**).

**Table 5:** Association between the TyG index and new-onset hypertension stratified by baseline fatty liver status.

Model	No fatty liver HR (95% CI), P value	Fatty liver HR (95% CI), P value	P for interaction
Unadjusted	2.40 (2.30-2.51), <0.001	1.27 (1.22-1.33), <0.001	<0.001
Model 1	1.68 (1.60-1.77), <0.001	1.18 (1.12-1.23), <0.001	<0.001
Model 2	1.46 (1.33-1.59), <0.001	1.22 (1.12-1.33), <0.001	<0.001

Values are presented as hazard ratios (HRs) and 95% confidence intervals (CIs), with corresponding P values. The outcome was new-onset hypertension. The interaction was tested by including a multiplicative interaction term between the TyG index and baseline fatty liver status.

Model 1 was adjusted for age and sex. Model 2 was adjusted for age, sex, marital status, ethnicity, body mass index, smoking status, alcohol drinking status, physical activity, total cholesterol, low-density lipoprotein cholesterol, high-density lipoprotein cholesterol, aspartate aminotransferase, and alanine aminotransferase. The sample size was 55,107 for the unadjusted model and Model 1. For Model 2, the sample sizes were 29,605 among participants without fatty liver and 12,379 among participants with fatty liver. Missing questionnaire responses for smoking status, alcohol drinking status, and physical activity were coded as an “unknown” category, whereas missing data for the other Model 2 covariates were handled using a complete-case approach. TyG, triglyceride-glucose index; HR, hazard ratio; CI, confidence interval.

## Discussion

In this large health-examination cohort of participants without hypertension at baseline, a higher TyG index and baseline fatty liver were each associated with an increased risk of new-onset hypertension. Participants with both high TyG and fatty liver had the highest risk. We also observed that baseline fatty liver modified the association between TyG and new-onset hypertension, with a stronger relative association among participants without fatty liver. These findings suggest that TyG-related glucose-lipid dysregulation and fatty liver are both associated with future hypertension risk, while fatty liver status may influence the relative association between TyG and new-onset hypertension.

The positive association between TyG and new-onset hypertension in our study is consistent with previous longitudinal and meta-analytic evidence. In a national cohort study of Chinese adults, higher TyG was associated with an increased risk of new-onset hypertension, with a positive dose-response relationship (16). A meta-analysis similarly reported a higher prevalence of hypertension among participants in the highest TyG category than among those in the lowest category (17). In addition, a longitudinal study of Mexican adults found that a higher TyG index was associated with incident hypertension during long-term follow-up (18). This association has also been observed in the United States, where a cross-sectional analysis of the 2001–2020 National Health and Nutrition Examination Survey showed that higher TyG was associated with higher odds of hypertension among adults without diabetes (19). Our findings extend this evidence by evaluating the TyG-hypertension association in relation to baseline fatty liver status within the same health-examination cohort. Taken together, evidence from Chinese, Mexican, and US populations, complemented by meta-analytic data, suggests that the TyG-hypertension association is not confined to a single population. Nevertheless, further studies in other ethnic groups and healthcare settings are needed to determine the consistency and transportability of this association.

Several biological mechanisms may explain the association between TyG and new-onset hypertension. TyG is derived from fasting triglycerides and fasting glucose and is widely used as a surrogate marker of insulin resistance (7). Insulin resistance and compensatory hyperinsulinemia may increase renal sodium reabsorption, promote volume expansion, activate the sympathetic nervous system, and impair endothelial function and vascular reactivity, thereby contributing to elevated blood pressure (20). In addition, a higher TyG index may reflect a broader state of glucose-lipid metabolic dysregulation that is associated with dyslipidemia, low-grade inflammation, oxidative stress, and arterial stiffness (20, 21). These pathways provide a plausible explanation for the observed association between higher TyG and subsequent hypertension.

Our finding that baseline fatty liver was associated with new-onset hypertension is also supported by previous evidence. A systematic review and meta-analysis reported a bidirectional association between nonalcoholic fatty liver disease and hypertension (22). Evidence from the UK Biobank further supports a temporal relationship between hepatic steatosis and blood pressure elevation. Hu et al. found that hepatic steatosis was more strongly related to subsequent blood pressure elevation than the reverse association (23). This finding was also supported by an analysis using MRI-derived liver fat measurements. More recently, an ultrasound-based cohort study of young adults found that fatty liver was associated with incident hypertension, with higher risks among those with more severe steatosis (24). In a cohort of participants with prehypertension, greater fatty liver severity was associated with progression to hypertension during follow-up (25). Together, evidence from the UK Biobank and other cohort and meta-analytic studies provides broader international support for fatty liver as a marker of increased future hypertension risk. However, differences in population composition, fatty liver definitions, and outcome ascertainment should be considered when comparing these findings.

Fatty liver may contribute to hypertension through several interrelated metabolic and vascular pathways. Rather than being an isolated imaging finding, fatty liver is increasingly regarded as a manifestation of systemic metabolic dysfunction characterized by hepatic insulin resistance, inflammatory activation, altered adipokine secretion, oxidative stress, and vascular dysfunction (26, 27). Previous evidence has linked fatty liver with impaired flow-mediated dilation and increased arterial stiffness, both of which are relevant to hypertension development (28, 29). Inflammation, activation of the renin-angiotensin-aldosterone system and sympathetic nervous system, and altered metabolic organ-secreted factors may further connect fatty liver-related metabolic dysfunction with vascular injury and elevated blood pressure (26, 27, 30).

Beyond the individual associations of TyG and fatty liver with new-onset hypertension, we observed that baseline fatty liver modified the association between TyG and new-onset hypertension. The relative association was stronger among participants without fatty liver than among those with fatty liver. Previous longitudinal studies have shown that a higher TyG index is associated with the development of fatty liver (31, 32), but they did not examine whether baseline fatty liver altered the association between TyG and incident hypertension. Our study extends this literature by evaluating fatty liver as a potential modifier of the TyG-hypertension association.

Several mechanisms may help explain this pattern. Recent literature on lean MASLD indicates that hepatic steatosis can occur in individuals with normal body weight and may coexist with insulin resistance and other metabolic abnormalities (33, 34), highlighting the metabolic heterogeneity of fatty liver that may not be fully captured by BMI. Consistent with this concept, the association between TyG and new-onset hypertension remained broadly similar across the evaluated BMI strata, suggesting that BMI did not materially modify the observed association. Among participants without fatty liver, an elevated TyG index may more clearly capture early glucose-lipid metabolic disturbance before hepatic steatosis is detectable by ultrasound. In contrast, among participants with fatty liver, hypertension risk may already be influenced by hepatic insulin resistance, inflammation, endothelial dysfunction, arterial stiffness, and other metabolic and vascular pathways (26–30). Consequently, the additional relative association of TyG with new-onset hypertension may therefore be less pronounced in this metabolically high-risk group. These potential explanations are speculative and should be confirmed in future mechanistic and prospective studies with repeated assessments of TyG, liver fat, and blood pressure.

The joint exposure analysis provides further context. Participants with both high TyG and fatty liver had the highest risk of new-onset hypertension. Thus, the weaker relative TyG-hypertension association among participants with fatty liver should be interpreted as effect modification within a group with a higher underlying metabolic and vascular risk burden, rather than as evidence that fatty liver lowers hypertension risk or that TyG is biologically unimportant in this group.

This study has several strengths. The large health-examination cohort provided sufficient statistical power to evaluate incident hypertension and to perform stratified analyses. Excluding participants with hypertension at baseline enabled the analysis to focus on new-onset hypertension during follow-up. In addition, the simultaneous evaluation of TyG, fatty liver, their joint association, and the modifying role of fatty liver provided a more integrated assessment of metabolic risk than evaluating either marker alone.

Several limitations should also be considered. First, although the cohort design established the temporal sequence from baseline TyG and fatty liver status to subsequent hypertension, the observational design does not permit causal inference. Although we additionally adjusted for family history of hypertension, baseline diabetes status, estimated glomerular filtration rate, baseline systolic blood pressure, and baseline diastolic blood pressure in sensitivity analyses, residual confounding cannot be excluded. Dietary sodium intake, detailed dietary patterns, sleep duration, socioeconomic status, antihyperglycemic medication use, lipid-lowering medication use, and genetic susceptibility were not fully measured. Second, TyG and fatty liver status were assessed only at baseline. Changes in metabolic status, liver fat, lifestyle factors, or medication use during follow-up were not fully captured. Third, blood pressure was assessed during scheduled health examinations using an automated device. Although the device was regularly calibrated and maintained, the exact biological onset of hypertension may have occurred between two examination visits. The event date was therefore defined as the first visit at which hypertension was detected, and no interval-censored model was used in the present analysis. Fourth, fatty liver was ascertained by abdominal ultrasonography during routine health examinations and analyzed as a binary exposure. Although ultrasonography is widely used in clinical practice, it is less sensitive for mild steatosis than magnetic resonance-based methods and cannot distinguish fatty liver etiologies or quantify liver fat severity. In addition, fatty liver status was obtained from existing clinical ultrasound reports, and the original images were not re-read for this study. Therefore, interobserver agreement could not be evaluated. Participants were not formally classified according to MASLD criteria. Therefore, our findings describe effect modification by ultrasonographic fatty liver and do not establish whether the TyG-hypertension association differs across specific MASLD phenotypes, including lean MASLD. Fifth, smoking, alcohol consumption, and physical activity were based on questionnaire data and may be subject to recall bias or misclassification. In addition, missing baseline covariate data were handled using unknown categories or a complete-case approach, which may have affected the estimates. Sixth, the analytic cohort was limited to participants with at least one subsequent health examination. Only 79,905 of 247,196 individuals (32.3%) underwent follow-up. Most measured clinical characteristics were broadly comparable between participants with and without follow-up, although the completeness of smoking status, alcohol consumption, physical activity, and baseline fatty liver data differed between the groups (**Supplementary Table 1**). Therefore, selection bias cannot be excluded even when the measured clinical characteristics appear broadly similar. The observed associations may be most applicable to adults undergoing repeat health examinations, and caution is warranted when extending the findings to individuals without follow-up or to other populations. Seventh, although the association between TyG and incident hypertension remained broadly consistent across BMI strata, BMI is an imperfect proxy for adiposity and does not capture central fat distribution or body composition. Therefore, the BMI-stratified analyses cannot establish that the observed association is fully independent of adiposity, and residual confounding by unmeasured adiposity cannot be excluded. Finally, the study population was derived from a health-examination setting, which may limit generalizability to community-based populations, high-risk clinical populations, or populations from other geographic and ethnic backgrounds.

## Conclusions

In conclusion, higher TyG index and baseline fatty liver were each associated with increased risk of new-onset hypertension, and participants with both high TyG and fatty liver had the highest risk. Baseline fatty liver status significantly modified the association between TyG and new-onset hypertension, with a stronger TyG-hypertension association observed among participants without fatty liver. These findings suggest that fatty liver status should be considered when interpreting the relationship between glucose-lipid metabolic disturbance, as reflected by TyG, and future hypertension risk.

## Data Availability

The data analyzed in this study is subject to the following licenses/restrictions: The datasets generated and/or analyzed during the current study are not publicly available due to institutional data protection policies but are available from the corresponding author on reasonable request. Requests to access these datasets should be directed to 1016099047@qq.com.
