# Supplementary material for: Effect modification by fatty liver in the association between triglyceride-Glucose index and new-onset hypertension: a large health-examination cohort study
Source: Front Endocrinol (Lausanne). 2026 Aug 31;17:1897050. doi: 10.3389/fendo.2026.1897050 (PMC13572287; doi:10.3389/fendo.2026.1897050)
Supplement: Supplementary file 1 [file Table1.docx]

**Supplementary Table S1**. Baseline characteristics of participants included in the analytic cohort and otherwise eligible individuals excluded because of the absence of follow-up examinations

| Characteristic | Included cohort (n = 66,664) | Excluded: no follow-up (n = 73,848) | P value | SMD |
| --- | --- | --- | --- | --- |
| Participants, n | 66,664 | 73,848 |  |  |
| Sex |  |  | <0.001 | 0.028 |
| Male | 30,314 (45.5%) | 34,625 (46.9%) |  |  |
| Female | 36,350 (54.5%) | 39,223 (53.1%) |  |  |
| Age, years | 43.8 ± 13.5 | 43.0 ± 13.3 | <0.001 | 0.060 |
| Marital status |  |  | <0.001 | 0.071 |
| Unmarried | 8,833 (13.3%) | 11,262 (15.3%) |  |  |
| Married | 57,720 (86.6%) | 62,307 (84.4%) |  |  |
| Missing | 111 (0.2%) | 279 (0.4%) |  |  |
| Ethnicity |  |  | <0.001 | 0.247 |
| Han | 58,516 (87.8%) | 58,068 (78.6%) |  |  |
| Non-Han | 630 (0.9%) | 903 (1.2%) |  |  |
| Missing | 7,518 (11.3%) | 14,877 (20.1%) |  |  |
| Systolic blood pressure, mmHg | 117.2 ± 12.3 | 118.1 ± 12.1 | <0.001 | 0.074 |
| Diastolic blood pressure, mmHg | 70.3 ± 8.8 | 70.9 ± 8.8 | <0.001 | 0.068 |
| Body mass index, kg/m² | 23.0 ± 3.1 | 23.3 ± 3.3 | <0.001 | 0.094 |
| Waist circumference, cm | 77.5 ± 8.9 | 78.5 ± 9.4 | <0.001 | 0.109 |
| Fasting glucose, mmol/L | 4.8 ± 0.9 | 4.9 ± 1.2 | <0.001 | 0.094 |
| Triglycerides, mmol/L | 1.5 ± 1.3 | 1.6 ± 1.4 | <0.001 | 0.074 |
| High-density lipoprotein cholesterol, mmol/L | 1.3 ± 0.3 | 1.3 ± 0.3 | 0.868 | 0.000 |
| Low-density lipoprotein cholesterol, mmol/L | 3.1 ± 0.9 | 3.1 ± 0.9 | <0.001 | 0.000 |
| Alanine aminotransferase, U/L | 26.5 ± 25.4 | 28.5 ± 33.2 | <0.001 | 0.068 |
| Aspartate aminotransferase, U/L | 24.1 ± 13.1 | 24.8 ± 20.7 | 0.987 | 0.040 |
| Serum creatinine, μmol/L | 63.7 ± 15.9 | 63.5 ± 16.7 | 0.010 | 0.012 |
| Urea, mmol/L | 5.4 ± 1.3 | 5.4 ± 1.4 | <0.001 | 0.000 |
| Smoking status |  |  | <0.001 | 0.647 |
| Never | 5,836 (8.8%) | 24,029 (32.5%) |  |  |
| Former | 37 (0.1%) | 164 (0.2%) |  |  |
| Current | 569 (0.9%) | 3,135 (4.2%) |  |  |
| Unknown/missing | 60,222 (90.3%) | 46,520 (63.0%) |  |  |
| Alcohol consumption |  |  | <0.001 | 0.646 |
| Never | 5,574 (8.4%) | 23,252 (31.5%) |  |  |
| Former | 27 (0.0%) | 106 (0.1%) |  |  |
| Current | 808 (1.2%) | 3,903 (5.3%) |  |  |
| Unknown/missing | 60,255 (90.4%) | 46,587 (63.1%) |  |  |
| Physical activity |  |  | <0.001 | 0.630 |
| None | 3,542 (5.3%) | 13,827 (18.7%) |  |  |
| Occasional | 2,515 (3.8%) | 11,992 (16.2%) |  |  |
| Regular | 166 (0.2%) | 444 (0.6%) |  |  |
| Unknown/missing | 60,441 (90.7%) | 47,585 (64.4%) |  |  |
| Baseline fatty liver status |  |  | <0.001 | 0.809 |
| No fatty liver | 38,294 (57.4%) | 21,980 (29.8%) |  |  |
| Fatty liver | 16,813 (25.2%) | 10,267 (13.9%) |  |  |
| Missing | 11,557 (17.3%) | 41,601 (56.3%) |  |  |

Values are presented as mean ± standard deviation or n (%). Percentages use the total number of participants in each column as the denominator. Continuous summaries are based on available data.

P values were calculated using independent-samples t tests or Wilcoxon rank-sum tests for continuous variables, and chi-square tests or Fisher exact tests for categorical variables, as appropriate.

Absolute SMDs are reported. For continuous variables, SMDs were calculated using the difference in means divided by the square root of the average of the two group variances. For categorical variables, SMDs were calculated across all displayed categories, including missing or unknown values.

Missing responses for smoking, alcohol consumption, and physical activity were treated as an unknown category in adjusted Cox models. Participants with missing baseline fatty liver status were excluded from analyses involving fatty liver; missing fatty liver status was not modeled as a third exposure category.

Abbreviation: SMD, standardized mean difference.
