# Supplementary material for: Effect modification by fatty liver in the association between triglyceride-Glucose index and new-onset hypertension: a large health-examination cohort study
Source: Front Endocrinol (Lausanne). 2026 Aug 31;17:1897050. doi: 10.3389/fendo.2026.1897050 (PMC13572287; doi:10.3389/fendo.2026.1897050)
Supplement: Supplementary file 2 [file Table2.docx]

**Supplementary Table S2.** Stratified associations of the triglyceride-glucose index with new-onset hypertension according to BMI category and baseline fatty liver status

| BMI category (kg/m²) | No fatty liver n (model N) | No fatty liver HR (95% CI), P value | Fatty liver n (model N) | Fatty liver HR (95% CI), P value | P for interaction |
| --- | --- | --- | --- | --- | --- |
| Underweight <18.5 | 3,016 (2,448) | 1.86 (1.10–3.16), 0.021 | 17 (13) | NE | <0.001 |
| Normal weight 18.5–23.9 | 28,047 (21,753) | 1.57 (1.40–1.76), <0.001 | 4,586 (3,306) | 1.27 (1.06–1.54), 0.012 |  |
| Overweight 24.0–27.9 | 6,774 (5,106) | 1.33 (1.14–1.55), <0.001 | 9,147 (6,749) | 1.24 (1.10–1.39), <0.001 |  |
| Obesity ≥28.0 | 412 (304) | 1.66 (0.91–3.05), 0.101 | 3,042 (2,313) | 1.21 (1.02–1.45), 0.030 |  |

Values are presented as hazard ratios (HRs) with 95% confidence intervals (CIs). HRs were estimated per 1-unit increase in the TyG index using Cox proportional hazards models.

BMI categories were defined according to Chinese adult cutoffs: underweight, <18.5 kg/m²; normal weight, 18.5–23.9 kg/m²; overweight, 24.0–27.9 kg/m²; and obesity, ≥28.0 kg/m².

Each BMI category was analyzed separately within participants with and without baseline fatty liver. Models were adjusted for sex, age, marital status, ethnicity, smoking, physical activity, total cholesterol, LDL-C, and HDL-C. BMI was not additionally adjusted within the BMI-stratified models.

P for interaction was obtained by adding the TyG × baseline fatty liver interaction term to the fully adjusted Cox proportional hazards model. This overall interaction P value is shown once and is not estimated separately within each BMI category.

NE, not estimable because of insufficient sample size or events in the underweight fatty liver subgroup.

Abbreviations: BMI, body mass index; CI, confidence interval; HR, hazard ratio; LDL-C, low-density lipoprotein cholesterol; NE, not estimable; TyG, triglyceride-glucose index.
