# Supplementary material for: Effect modification by fatty liver in the association between triglyceride-Glucose index and new-onset hypertension: a large health-examination cohort study
Source: Front Endocrinol (Lausanne). 2026 Aug 31;17:1897050. doi: 10.3389/fendo.2026.1897050 (PMC13572287; doi:10.3389/fendo.2026.1897050)
Supplement: Supplementary file 3 [file Table3.docx]

**Supplementary Table S3.** Sensitivity analyses additionally adjusted for family history of hypertension, baseline diabetes status, and estimated glomerular filtration rate

Panel A. Association between TyG and new-onset hypertension

| TyG variable | Model 3 HR (95% CI) | P value |
| --- | --- | --- |
| TyG, continuous | 1.42 (1.34–1.51) | <0.001 |
| Q1 (≤8.16) | Ref |  |
| Q2 (>8.16 to ≤8.557) | 1.31 (1.22–1.40) | <0.001 |
| Q3 (>8.557 to ≤8.989) | 1.50 (1.40–1.61) | <0.001 |
| Q4 (>8.989) | 1.65 (1.52–1.80) | <0.001 |

Model sample size: 50,199.

Panel B. Association between baseline fatty liver status and new-onset hypertension

| Baseline fatty liver status | Model 3 HR (95% CI) | P value |
| --- | --- | --- |
| No fatty liver | Ref |  |
| Fatty liver | 1.26 (1.19–1.33) | <0.001 |

Model sample size: 41,959.

Panel C. Joint association of TyG and fatty liver status with new-onset hypertension

| Joint exposure group | Model 3 HR (95% CI) | P value |
| --- | --- | --- |
| Low TyG + no fatty liver | Ref |  |
| Low TyG + fatty liver | 1.45 (1.31–1.60) | <0.001 |
| High TyG + no fatty liver | 1.42 (1.32–1.52) | <0.001 |
| High TyG + fatty liver | 1.64 (1.52–1.76) | <0.001 |

Model sample size: 41,959.

Panel D. Association between TyG and new-onset hypertension stratified by baseline fatty liver status

| Baseline fatty liver status | Model 3 HR (95% CI) | P value | P for interaction |
| --- | --- | --- | --- |
| No fatty liver | 1.50 (1.36–1.64) | <0.001 | <0.001 |
| Fatty liver | 1.21 (1.10–1.33) | <0.001 |  |

Model sample size: 41,959.

Table note: Model 3 was additionally adjusted for family history of hypertension, baseline diabetes status, and estimated glomerular filtration rate, in addition to age, sex, marital status, ethnicity, body mass index, smoking status, alcohol drinking status, physical activity, total cholesterol, low-density lipoprotein cholesterol, high-density lipoprotein cholesterol, aspartate aminotransferase, and alanine aminotransferase.
In Panel D, multiplicative interaction was assessed using a TyG-by-fatty liver interaction term. Low and high TyG in Panel C were defined using the receiver operating characteristic curve-derived cutoff used in the primary joint analysis. TyG, triglyceride-glucose index. HR, hazard ratio. CI, confidence interval. Ref, reference.
