# Supplementary material for: Effect modification by fatty liver in the association between triglyceride-Glucose index and new-onset hypertension: a large health-examination cohort study
Source: Front Endocrinol (Lausanne). 2026 Aug 31;17:1897050. doi: 10.3389/fendo.2026.1897050 (PMC13572287; doi:10.3389/fendo.2026.1897050)
Supplement: Supplementary file 4 [file Table4.docx]

**Supplementary Table S4.** Sensitivity analyses additionally adjusted for family history of hypertension, baseline diabetes status, estimated glomerular filtration rate, baseline systolic blood pressure, and baseline diastolic blood pressure.

Panel A. Association between TyG and new-onset hypertension

| TyG variable | Model 4 HR (95% CI) | P value |
| --- | --- | --- |
| TyG, continuous | 1.08 (1.02-1.15) | 0.008 |
| Q1 (<=8.16) | Ref |  |
| Q2 (>8.16 to <=8.557) | 1.16 (1.08-1.24) | <0.001 |
| Q3 (>8.557 to <=8.989) | 1.19 (1.11-1.28) | <0.001 |
| Q4 (>8.989) | 1.17 (1.08-1.28) | <0.001 |

Model sample size: 50,055.

Panel B. Association between baseline fatty liver status and new-onset hypertension

| Baseline fatty liver status | Model 4 HR (95% CI) | P value |
| --- | --- | --- |
| No fatty liver | Ref |  |
| Fatty liver | 1.12 (1.06-1.19) | <0.001 |

Model sample size: 41,843.

Panel C. Joint association of TyG and fatty liver status with new-onset hypertension

| Joint exposure group | Model 4 HR (95% CI) | P value |
| --- | --- | --- |
| Low TyG + no fatty liver | Ref |  |
| Low TyG + fatty liver | 1.27 (1.14-1.40) | <0.001 |
| High TyG + no fatty liver | 1.20 (1.12-1.28) | <0.001 |
| High TyG + fatty liver | 1.27 (1.18-1.37) | <0.001 |

Model sample size: 41,843.

Panel D. Association between TyG and new-onset hypertension according to baseline fatty liver status

| Baseline fatty liver status | Model 4 HR (95% CI) | P value | P for interaction |
| --- | --- | --- | --- |
| No fatty liver | 1.13 (1.03-1.24) | 0.009 | <0.001 |
| Fatty liver | 1.00 (0.91-1.10) | 0.972 |  |

Model sample size: 29,519 among participants without fatty liver and 12,324 among participants with fatty liver.

Model 4 was adjusted for age, sex, marital status, ethnicity, body mass index, smoking status, alcohol drinking status, physical activity, total cholesterol, low-density lipoprotein cholesterol, high-density lipoprotein cholesterol, aspartate aminotransferase, alanine aminotransferase, family history of hypertension, baseline diabetes status, estimated glomerular filtration rate, baseline systolic blood pressure, and baseline diastolic blood pressure.
TyG, triglyceride-glucose index; HR, hazard ratio; CI, confidence interval.
