# Supplementary material for: Effect modification by fatty liver in the association between triglyceride-Glucose index and new-onset hypertension: a large health-examination cohort study
Source: Front Endocrinol (Lausanne). 2026 Aug 31;17:1897050. doi: 10.3389/fendo.2026.1897050 (PMC13572287; doi:10.3389/fendo.2026.1897050)
Supplement: Supplementary file 5 [file Table5.docx]

**Supplementary Table S5.** Additive interaction between high TyG and baseline fatty liver status in relation to new-onset hypertension

| Model | RERI (95% CI) | AP (95% CI) | SI (95% CI) |
| --- | --- | --- | --- |
| Unadjusted | −0.21 (−0.52 to 0.09) | −0.05 (−0.13 to 0.02) | 0.93 (0.84 to 1.03) |
| Model 1 | −0.18 (−0.38 to 0.03) | −0.08 (−0.16 to 0.01) | 0.88 (0.76 to 1.01) |
| Model 2 | −0.21 (−0.43 to 0.01) | −0.13 (−0.26 to 0.00) | 0.76 (0.55 to 0.97) |

RERI, relative excess risk due to interaction; AP, attributable proportion due to interaction; SI, synergy index; CI, confidence interval. Additive interaction measures were calculated on the hazard ratio scale using the low TyG plus no fatty liver group as the reference category. High TyG was defined as a TyG index >8.4, based on the receiver operating characteristic curve-derived cutoff. RERI >0, AP >0, or SI >1 indicates positive additive interaction. Model 1 was adjusted for age and sex. Model 2 was adjusted for age, sex, marital status, ethnicity, body mass index, smoking status, alcohol drinking status, physical activity, total cholesterol, low-density lipoprotein cholesterol, and high-density lipoprotein cholesterol.
